# Glomerular changes and alterations of zonula occludens-1 in the kidneys of Plasmodium falciparum malaria patients

**DOI:** 10.1186/1475-2875-13-176

**Published:** 2014-05-09

**Authors:** Benjamas Wichapoon, Chuchard Punsawad, Urai Chaisri, Parnpen Viriyavejakul

**Affiliations:** 1 Department of Tropical Pathology, Faculty of Tropical Medicine, Mahidol University, 420/6 Rajvithi Road, Bangkok 10400, Thailand; 2 School of Medicine, Walailak University, 222 Thasala District, Nakhon Si Thammarat 80161, Thailand

**Keywords:** Zonula occludens-1, ZO-1, Tight junction, Kidney, Glomeruli, *Plasmodium falciparum*, Acute kidney injury, AKI

## Abstract

**Background:**

The process of cytoadhesion in *Plasmodium falciparum* malaria infection causes signaling processes that lead to structural and functional changes at the cellular level. Histopathological changes of acute kidney injury (AKI) in *P. falciparum* malaria often involve glomerular proliferation, thickening of the glomerular basement membrane, acute tubular necrosis, and interstitial inflammation. Focusing on the glomeruli, this study aimed to investigate glomerular and tight junction-associated protein- zonula occludens-1 (ZO-1) changes in *P. falciparum* malaria patients.

**Methods:**

Kidney tissues were grouped into *P. falciparum* with AKI (Cr ≥ 265 μmol/L or 3 mg/dl), *P. falciparum* without AKI (Cr < 265 μmol/L), and normal kidney tissues (control group). Glomerular cells and the glomerular area were quantified and compared in three experimental groups. The tight junction was investigated immunohistochemically using tight junction-associated protein, ZO-1, protein marker. A further immunofluorescence study was performed in an endothelial cell (EC)-parasitized red blood cell (PRBC) co-culture system, to evaluate the tight junction protein.

**Results:**

Glomerular cell proliferation was significant in *P. falciparum* with AKI (Cr ≥ 265 μmol/L). By contrast, the glomerular area decreased significantly. ZO-1 expression was significantly decreased in the AKI group compared with normal kidneys, and in kidney tissues without AKI (*p* < 0.05). This was further confirmed by the depletion in ZO-1 localization in ECs co-cultured with PRBCs.

**Conclusions:**

In *P. falciparum* malaria with AKI, the decrease in glomerular area, despite glomerular cell proliferation, could be due to the collapse of cellular structures secondary to damaged tight junction-associated protein, ZO-1.

## Background

Malaria infection caused by *Plasmodium falciparum* is the world’s most widespread infection. Among its severe manifestations, acute kidney injury (AKI) is an important complication, which can cause mortality of up to 45% [1]. The overall incidence of AKI has been reported as between 1-4.8% in endemic areas, and 25-30% in non-immune patients [2]. Malarial complications are possibly caused by the interaction of the parasite with the host, resulting in mechanical, immunologic, and humoral responses. Responses to eliminate malaria parasites may also injure host tissues [3]. The precise mechanism of AKI in *P. falciparum* malaria is not fully understood. Several hypotheses proposed for the pathogenesis of AKI in malaria include mechanical obstruction by parasitized red blood cells (PRBCs), exaggerated host immune response mediated through cytokines, reactive oxygen and nitrogen species, immune complex deposition, hypovolaemia, and disturbances in the renal microcirculation [4]. Changes in the glomeruli often include glomerular cell proliferation, basement membrane thickening, presence of PRBCs and protein deposition within the mesangium, endothelial cell wall, and Bowman’s capsule [3]. The process of cytoadhesion of PRBCs to the vascular endothelial cells (ECs) of different host organs, along with rosette formation, is considered the most important mechanism of severe *P. falciparum* malaria [5]. ECs located in the capillary loop of the glomeruli provide target adhesion sites for PRBCs. This leads to the disruption of the EC barrier and eventually affects vascular permeability. Tight junctions are located apical to the EC surface and composed of multiple transmembrane, scaffolding, and signaling proteins. There are at least three different types of transmembrane proteins within tight junctions: occludin, claudins, and junctional adhesion molecule (JAM) [6]. Zonula occludens-1 (ZO-1) proteins are found associated with the cytoplasmic surfaces of tight junctions [7,8]. They are in contact with the extracellular tight junction membrane proteins and provide a link between the integral membrane proteins and the filamentous cytoskeleton. In addition to the evaluation of glomerular changes in the kidney of *P. falciparum* patients, this study focused on structural alterations to the junction-associated protein, ZO-1, in *P. falciparum* malaria.

## Methods

### Tissue specimens

Kidney tissues from fatal *P. falciparum* cases were classified into *P. falciparum* with acute kidney injury (AKI) (creatinine, (Cr) ≥ 265 μmol/L or 3 mg/dl), and *P. falciparum* without AKI (Cr < 265 μmol/L), based on Cr level before death. Control kidney tissues were obtained from stored specimens collected from accident fatalities with no kidney pathology, showing normal glomeruli, basement membrane, and tubules. The use of specimens and the research protocol were approved by the Ethics Committee of the Faculty of Tropical Medicine, Mahidol University (MUTM 2011-018-01, MUTM 2011-018-02). The pertinent clinical data documented from patient’s charts included sex, age, days of fever, admission parasite count, last parasite count, admission blood urea nitrogen (BUN) level, last BUN level, admission serum Cr, last serum Cr, day of hospitalization, and other complications.

### Histopathological preparation

All tissue were fixed in 10% buffered formalin for at least 24-48 hrs, then were subjected to standard histological processing for paraffin-embedded section. Kidney tissues were sectioned at 4 μm in thickness for histopathological and immunohistochemical studies. To evaluate the glomerular changes, kidney tissues were stained with Mayer’s haematoxylin and eosin (H&E). All cells within the glomeruli (mesangial cells, endothelial cells (ECs) and podocytes) were quantified based on morphology, by randomization from 30 glomeruli in a patient under 400x magnification. Mesangial cells are found inside the mesangium. ECs are localized inside the glomerular capillaries, in close contact with the capillary wall. Cells located outside, but in direct contact with the capillary wall, are considered podocytes [9]. In addition, the glomerular area was measured and analysed by the computer software program, “Image J analysis” [10], at a magnification of 100x. The slides were read blind to the clinical details of the patients by two persons (PV and BW).

### Immunohistochemical study of ZO-1

The expression of ZO-1 was detected by avidin-biotin peroxidase complex technique (Vector Laboratories, Inc., Burlingame, CA) according to the manufacturer’s protocol. Before immunohistochemical staining, the tissue sections were heated at 56.5°C, then deparaffinized and hydrated through xylene and graded alcohol series. All tissue sections were blocked for endogenous peroxidase activity and incubated for 30 mins in 3% H_2_O_2_ diluted in distilled water. The slides were then washed 4 times with Tris-buffer saline (TBS), at pH 7.4, for 5 mins. To induced epitope retrieval, the tissue sections were heated with Tris-HCl buffer (pH 9.0) by microwave oven at 800 W for 20 mins. After cooling to room temperature (RT), the slides were washed in TBS and were blocked with diluted normal goat serum for 30 mins to reduce the non-specific background. Then, the sections were incubated with primary antibody; rabbit polyclonal anti-ZO-1 (Invitrogen Corporation, Frederick, MD) (1:50 dilution) in blocking solution and incubated overnight at 4°C. The following day, the sections were washed 4 times in TBS, and incubated with diluted biotinylated secondary antibody; biotinylated goat anti-rabbit immunoglobulin (Vector Laboratories, Inc., Burlingame, CA) for 30 mins at RT to detect polyclonal antibody, and reacted with avidin-biotin complex (ABC) method conjugated with horseradish peroxidase (HRP) (Dako Laboratories, Inc., Glostrup, Denmark) for 30 mins. After washing, the peroxidase reaction was visualized with 0.05% 3,3’ diaminobenzidine (DAB) and 0.01% H_2_O_2_ for 10 mins, resulting in the formation of a brown reaction product at the antigen sites. Subsequently, the sections were counterstained with haematoxylin for 1 min. The slides were rinsed in running tap water for 10 mins, dehydrated, cleared, mounted with a cover slip and evaluated under a light microscope.

### Evaluation of immunohistochemistry staining

The proportions of immunopositive cells stained for ZO-1 were assessed. The expression of ZO-1 is specific for tight junction-associated protein [11] and stained brown in the glomeruli. Each slide was observed on ten different representative microscopic fields for immunopositive cells stained for the target marker. Consequently, the percentage of positive cells in each field was calculated compared with the number of total cells, and the average number of positive cells. Staining intensity was graded on a scale of 0 to 3, according to semi-quantitative assessment, as follows: 0 = no detectable staining, 1 = weakly positive, 2 = moderately positive, and 3 = strongly positive. The total score (TS) was calculated from the product of the percentage of immunopositive cells (%) and intensity (I) [Total score (TS) = percentage of positive cells (%) × intensity (I)], according to previous studies [12,13].

### Immunofluorescence study of tight junction (ZO-1) in ECs co-cultured with PRBCs

#### Preparation of ECs

To further investigate alterations in tight junction, an EC-malaria parasite model was used in the study. Human umbilical vein endothelial cells (HUVEC) were cultured from passage 4-6 (a gift from Prof. Steve Ward, Liverpool School of Tropical Medicine, UK). When HUVEC reached 80-90% confluence (in T25 flask), cells were trypsinized, seeded on 35 mm diameter gelatin-coated coverslip (Bioptechs, Butler, PA) and placed in a 40 mm diameter Petri dish. Approximately 500 μl of cell suspension were gently loaded onto the coated coverslips at a seeding density of 15,000-20,000 cells/cm^2^. Cells were allowed to settle for 1 hr at 37°C, 5% CO_2_, in a humidified incubator. The EC medium was then removed and 3 ml of pre-warmed fresh medium were added. Cells were then further cultured on the coverslip until they reached 80-90% confluence and were suitable for co-culture with PRBCs.

#### Preparation of PRBCs

*Plasmodium falciparum* strain ITG (a gift from Prof. Alister Craig, Liverpool School of Tropical Medicine, UK) was cultured to 5-8% parasitaemia (trophozoite or schizont stages).

#### Co-culture of ECs with PRBCs

The concentrated parasites were re-suspended in 1% fetal calf serum (FCS) in medium 199 to a parasitaemia of 50% in 1% haematocrit. EC medium was removed from the attached ECs (80-90% confluence) and the cells were gently washed once with 1X phosphate buffered saline solution (PBS). Parasite suspension (3 ml) was then added to the HUVEC monolayer. The co-culture dishes were gently agitated either clockwise or counter clockwise every 10-15 mins for 1 hr to allow more cell-to-cell contact, and co-cultured for 24 hr at 37°C, 5% CO_2_, in a humidified incubator. Control groups consisted of ECs grown on media alone and ECs cultured with red blood cell (RBC) suspension prepared to a haematocrit of 1% (medium 199 and 1% FCS).

#### Immunofluorescence staining of ZO-1

After 24 hrs of co-culture, PRBCs, RBCs and the media were gently removed from the HUVEC monolayer and washed gently with 1X PBS. The remaining attached EC monolayer was fixed with fresh 4% paraformadehyde in PBS for 15 mins, then rinsed with EC media. The ECs were gently washed 2 times with 1X PBS and permeabilized for 5 mins in RT with 2% Triton X-100 in 1X PBS. Cells were labeled with Anti-ZO-1 tight junction protein (Zymed Laboratories Inc. Laboratories, South San Francisco, CA) (1: 100 dilution) at 37°C, incubated for 1 hr, then washed 3 times with 1X PBS. An AlexaFluor®-conjugated goat anti-rabbit polyclonal IgG (Jackson Immunoresearch, West Grove, PA) (1: 400 dilution) was used as the secondary antibody. The ECs were incubated for 45 mins at 37°C, then the cells were washed 3 times with 2% Triton X-100 in 1X PBS. Nuclei were stained with DAPI (Invitrogen, Carlsbad, CA) (1 μg/ml; 5000X) in 1X PBS for 2 mins then washed once in 1X PBS. A small drop of mounting solution with anti-fade solution (Vector Laboratories, Burlingame, CA) was added to each slide and covered gently with a coverslip to prevent air bubbles. The cells were left for 15-30 mins at RT and sealed with nail polish.

#### Evaluation of ZO-1

Tight junction-associated protein, ZO-1, was considered altered when ZO-1 expression was depleted or the cell junction lacked continuity. ECs with damaged cell junctions were counted randomly in 300-450 cells and expressed as percentages.

### Statistical analysis

Data were analysed using descriptive statistics. All quantitative data were presented as mean ± standard error of mean (SEM). Normality of distribution was tested by Kolmogorov-Smirnov test. Comparison of difference in the mean, SEM, was calculated by one-way analysis of variance (ANOVA). The Pearson’s correlation test was used to estimate the strength and direction of the correlation between the expression of ZO-1 and the clinical data. Statistical analysis was performed using SPSS version 14.0 software (SPSS, Chicago, IL). Statistical significance was set at *p*-value ≤ 0.05.

## Results

### Clinical and laboratory data of malaria patients

**Figure 1:**
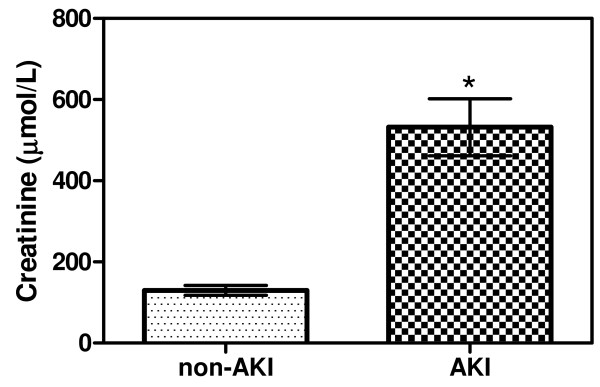
**Creatinine levels in fatal malaria patients.** A significant difference was observed between the two groups (*p* = 0.010). Data presented as mean ± SEM.

In this study, kidney specimens were obtained from 20 patients who died from *P. falciparum* malaria, comprising of 10 cases with acute kidney injury (AKI), and 10 cases without AKI. The clinical features of the patients are shown in Table 1. Serum creatinine (Cr) values of the two groups are shown in Figure 1. Parasitaemia and length of fever were similar in the two groups. Other complications in the AKI cases included cerebral malaria (6 of 10, 60%), pneumonia (2 of 10, 20%), jaundice (2 of 10, 20%), acute respiratory distress syndrome (ARDS) (1 of 10, 10%) and gastrointestinal bleeding (1 of 10, 10%).

**Figure 2:**
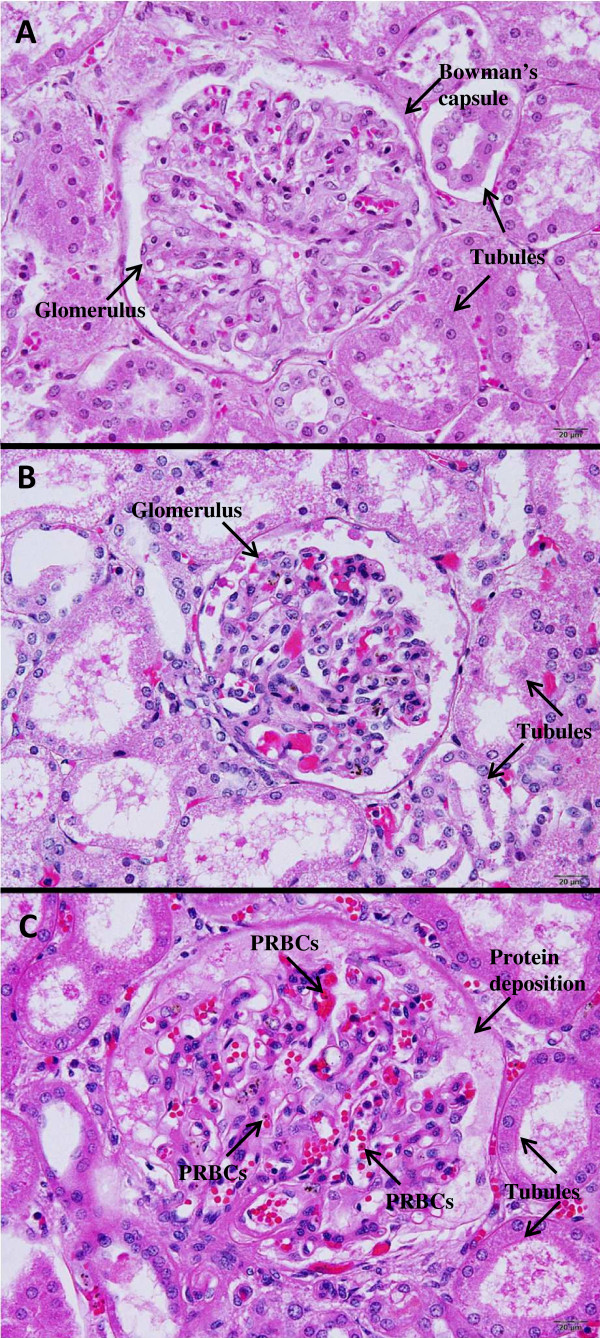
**Representative histopathology of glomeruli in the kidneys of the *P. falciparum *malaria patients, and the control group.** A normal kidney showing unremarkable glomerulus and intact tubules is shown in **(A)**. **(B)** and **(C)** illustrate glomeruli from *P. falciparum* malaria patients. Glomerular proliferation, protein deposition, and sequestration of PRBCs within the glomerular capillaries, are features of AKI in malaria **(C)**. All images are 400x magnification. Bars = 20 μm.

**Table 1:** Demographic and clinical data of the malaria patients

	**non-AKI (n = 10)**	**AKI (n = 10)**
Age	27.9 ± 5.5	27.3 ± 4.1
Sex (M:F)	9:1	6:4
Days of fever	14.99 ± 0.57	19.88 ± 1.44
Parasitaemia (on admission) (/μl)	379,813 ± 183,575	285,666 ± 90,400
Parasitaemia (last) (/μl)	32,897 ± 31,971	54,121 ± 51,804
BUN (on admission) (mmol/L)	14.46 ± 3.87	41.99 ± 4.54^*^
BUN (last) (mmol/L)	13.66 ± 2.54	32.53 ± 5.40^*^
Cr (on admission) (μmol/L)	124.74 ± 11.83	532.17 ± 70.73^*^
Cr (last) (μmol/L)	168.84 ± 30.62	432.44 ± 75.91^*^

^*^Significant difference of *p* < 0.05, compared with non-AKI (Cr < 265 μmol/L).

### Glomerular changes in P. falciparum malaria

#### Evaluation of glomerular cells

**Figure 3:**
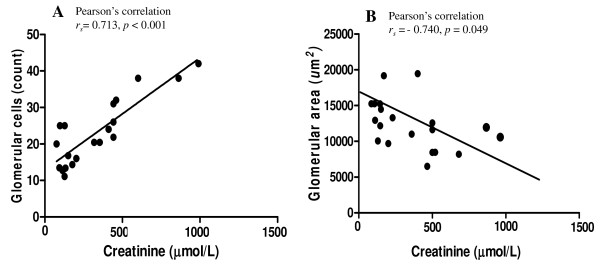
Correlation between glomerular cells and serum Cr level (A) and between glomerular area and serum Cr level (B).

The histopathology of kidney tissues from the *P. falciparum* malaria patients is illustrated in Figure 2. Glomerular proliferation was evaluated morphologically by quantification of mesangial cells, endothelial cells (ECs), and podocytes in the control, non-AKI, and AKI groups (Table 2). Glomerular cells were prominent in the AKI group. Other changes included protein deposition in the Bowman’s capsule, thickening of basement membrane congestion, and the presence of PRBCs within the capillaries. The total number of glomerular cells was significantly increased in AKI, compared with control and non-AKI kidney samples (*p* = 0.032, *p* = 0.041, respectively). No difference was observed between the non-AKI and control groups (*p* = 0.085). There was a significant positive correlation between total glomerular cell count in the kidney and serum Cr level (Pearson’s correlation *r*_*s*_ = 0.713, *p* < 0.001) (Figure 3A).

**Table 2:** Glomerular cells in the kidney of control, non-AKI (Cr < 265 μmol/L), and AKI (Cr ≥ 265 μmol/L), groups

**Glomerular cell types in a glomerulus**	**Control (n = 10)**	**non-AKI (n = 10)**	**AKI (n = 10)**
Mesangial cells	11.91 ± 0.22	12.63 ± 0.28	19.28 ± 1.67^*^’^**^
Endothelial cells	66.24 ± 4.40	81.23 ± 2.49	88.13 ± 4.21^*^’^**^
Podocytes	12.04 ± 0.98	14.99 ± 0.57	19.88 ± 1.44^*^’^**^
Total glomerular cells	30.06 ± 5.72	36.02 ± 7.16	42.43 ± 7.24^*^’^**^

^*^Significant difference of *p* < 0.05, compared to controls.

^**^Significant difference of *p* < 0.05, compared with non-AKI (Cr < 265 μmol/L).

Data presented as mean ± SEM.

#### Evaluation of glomerular area

**Figure 4:**
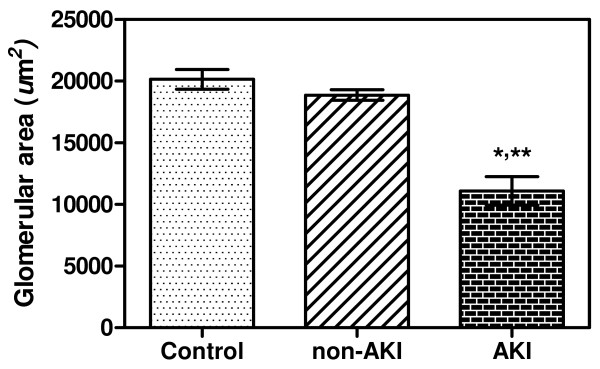
**Glomerular area (μm**^**2**^**) in the kidneys of *P. falciparum *malaria patients and controls.** Significant differences were observed between AKI and control groups (*p < 0.001), and between AKI and non-AKI group (**p < 0.001). Data presented as mean ± SEM.

The mean glomerular areas in the control, non-AKI, and AKI groups, were 20,151.09 ± 798.34 μm^2^, 18,872.02 ± 424.72 μm^2^, and 11,097.24 ± 1,160.45 μm^2^, respectively (Figure 4). The glomerular area was significantly decreased in AKI, compared with control kidney samples and non-AKI cases (all *p* < 0.001). There was a significant negative correlation between glomerular area and serum Cr level (Pearson’s correlation *r*_*s*_ = −0.740, *p* = 0.049) in the AKI and non-AKI cases (Figure 3B). No significant association was noted between glomerular area and other clinical data (sex, age, day of fever, admission parasite count, last parasite count, admission BUN level, last BUN level, day of hospitalization, or other complications.

#### Expression of ZO-1

**Figure 5:**
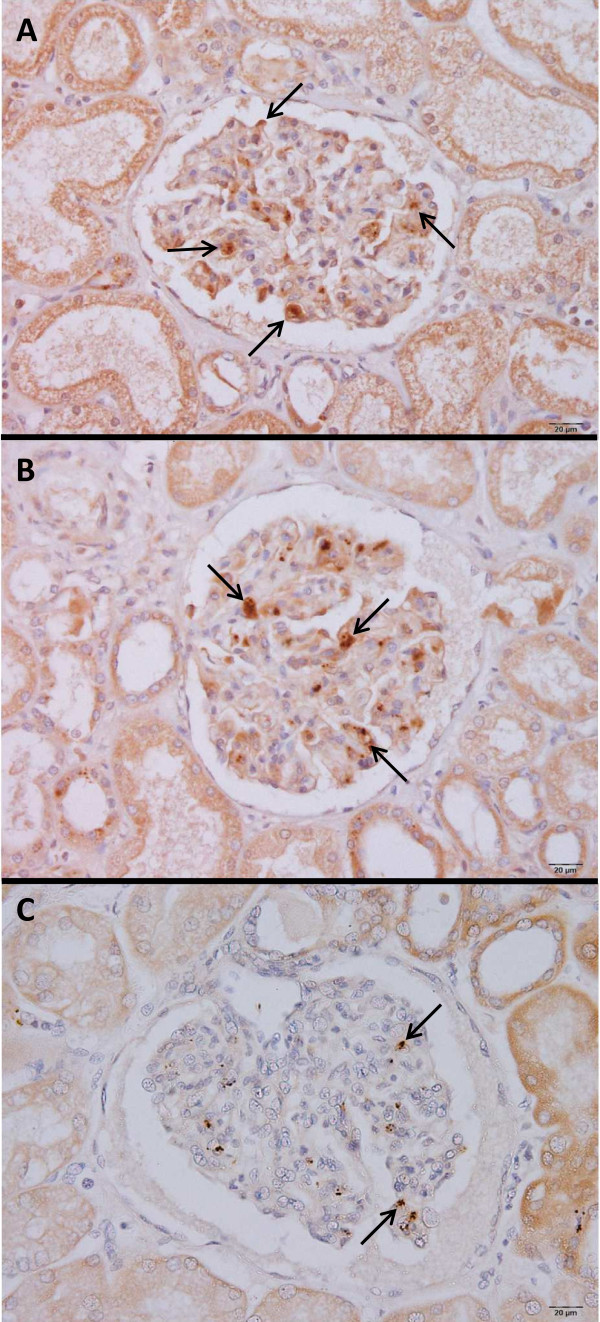
**Pattern of immunohistochemical staining for ZO-1 in the kidney tissues of control (A), non-AKI (B), and AKI (C) cases.** Arrows show positive staining for ZO-1. All images are 400x magnification. Bars = 20 μm.

**Figure 6:**
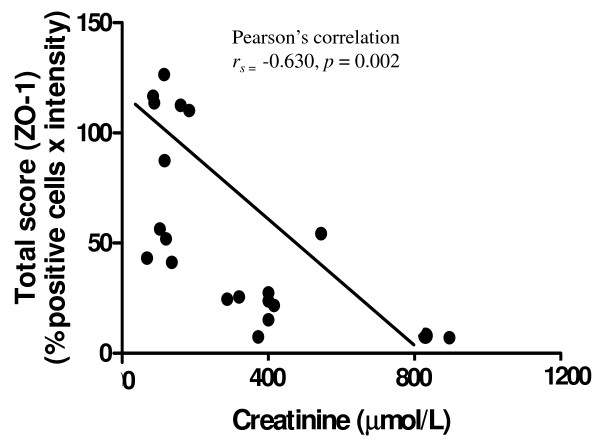
**Correlation between mean total score (% positive cells x intensity) of ZO-1 expression in the kidneys of *P. falciparum *** **malaria (n = 20), and serum Cr level.**

Expression of scaffolding tight-junction protein, ZO-1, was detected in the glomeruli, specifically ECs and foot process junctions. In the kidney tissues of the AKI cases, weak staining was observed in the glomeruli, compared with non-AKI and control kidney samples (Figure 5). A significant negative correlation was found between mean total score (% positive cells x intensity) of ZO-1 and serum Cr level (Pearson’s correlation *r*_*s*_ = −0.630, *p* = 0.002) in AKI and non-AKI cases (Figure 6).

#### Evaluation of ZO-1 using ECs-PRBCs model

**Figure 7:**
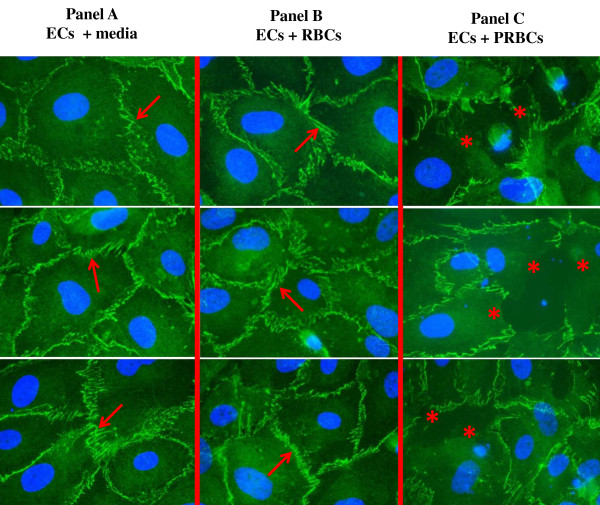
**ZO-1 expression in cell junctions of EC-PRBC co-culture.** Confluent EC monolayers were exposed to media alone (control), RBCs, or PRBCs for 24 hrs. After incubation, the ECs were stained with primary antibodies directed against ZO-1, followed by Alexafluor 488-conjugated secondary antibodies. Intact tight junction-associated protein is shown in normal ECs (incubated with medium alone) **(Panel A)** and ECs incubated with RBCs **(Panel B)**. Both panels **(A, B)** display continuity of ZO-1 expression along the cell membrane with no alteration (arrows). Damaged tight-junction-associated protein, ZO-1, is seen in ECs incubated with PRBCs **(Panel C)** (asterisks) (1,000x). Green- ZO-1, Blue- nuclei.

**Figure 8:**
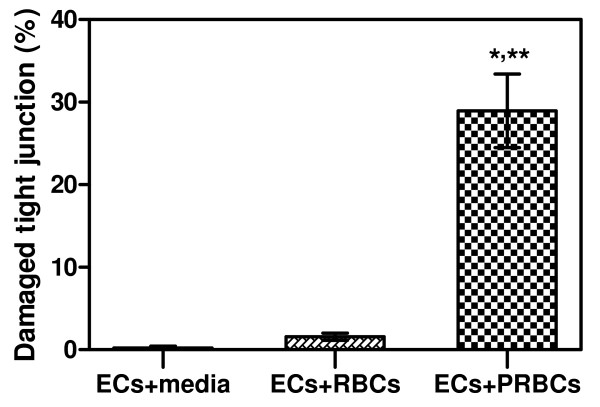
**Percentages of damaged tight-junction-associated protein, ZO-1 in normal ECs, ECs incubated with RBCs and ECs incubated with PRBCs.** Significant differences were observed between AKI and control groups (*p* = 0.003), and between AKI and non-AKI group (*p* = 0.004). Data presented as mean ± SEM.

Complex tight-junction-associated protein was further investigated by immunofluorescence study of ZO-1 in ECs co-cultured with PRBCs. Cell junction alteration was considered when EC cell membrane discontinuity and/or damaged cell junction were evident. ZO-1 was localized along the cell junction of the adherent EC monolayer, at the site of cell-to-cell contact. In the control ECs, and ECs incubated with RBCs, ZO-1 appeared continuously along the cell membrane (Figure 7, Panels A and B). A significant loss of ZO-1 expression in ECs co-culture with PRBCs (Figure 7, Panel C) was observed. Quantitatively, the percentages of damaged ZO-1 were significantly higher in ECs incubated with PRBCs (28.95% ± 4.47) than the control groups (ECs in media alone, 0.23% ± 0.22, *p* = 0.003), and ECs co-cultures with RBCs (1.59 ± 0.43, *p* = 0.004) (Figure 8).

## Discussion

In the AKI (Cr ≥ 265 μmol/L) group, histopathological changes in the glomeruli included proliferation of glomerular cells, protein deposition in the glomerular space, and decreased in the glomerular area, aside from the presence of parasitized red blood cells (PRBCs) inside the capillary lumen and marked congestion. Tubular cell changes showed cloudy swelling, a certain degree of tubular degeneration, and tubular necrosis. Chronic interstitial inflammation, consisting mainly of lymphocytes, was an important feature found in AKI. The thickening of the glomerular basement membrane was observed occasionally, which is similar to previous reports [2,4]. Protein deposition localized in the glomerular space could be immune complex deposits containing IgG, C3, and malarial antigens [3]. Glomerular cells, namely mesangial cells, ECs and podocytes, in the AKI were quantified based on morphology. The results showed a significant increase in glomerular cells in AKI kidneys with *P. falciparum* malaria compared with normal kidneys and non-AKI, consistent with previous studies [2,14-16]. In addition, the present study demonstrated a significant positive correlation between glomerular cells (mesangial cells, ECs, and podocytes) and serum Cr level, indicating that high levels of serum Cr affects glomerular cell proliferation in the kidneys of malaria patients. Proliferation of the glomerular cells is also a prominent feature of glomerular disease, including IgA nephropathy, membrano-proliferative glomerulonephritis, lupus nephritis, and diabetic nephropathy [17]. Glomerular cell proliferation may be due to the immune response secondary to cytokine release during malaria infection, by both host immune cells and malaria parasites. However, the glomerular area does not correlate with glomerular cell proliferation. The glomerular area was significantly reduced in AKI compared with non-AKI and normal kidneys, in contrast with the finding of glomerular cell proliferation. This can possibly be explained by glomerular constriction as a consequence of the damaged tight junction-associated protein, ZO-1 in AKI. The loss of tight junction-associated protein was demonstrated by the decrease in ZO-1 expression in the immunohistochemical study, and the damaged cell junction was illustrated by the immunofluorescence study. The alteration of ZO-1 may induce collapse of protein structures of EC cellular junctions. Subsequently, this causes glomerular cell contraction, which contributes to the decrease in glomerular area in malarial AKI. In addition, a negative correlation was seen between glomerular area and serum Cr level in AKI cases.

Tight-junction permeability may be regulated directly through modification of tight-junction proteins, or indirectly through effects on the cytoskeleton. Associated protein tight junction ZO-1 is a scaffold protein that provides the structural basis at the cytoplasmic surface of intercellular junctions, and localizes specifically at tight junctions [18,19]. ZO-1 is the most widely studied tight junction protein and plays an important role in stabilizing the tight junction complex structure [20] and signaling transduction [21]. In addition, ZO-1 is localized between foot processes in the glomerulus [22]. The ZO-1 protein possibly connects the slit diaphragm, indirectly or directly, to the cytoskeleton of a cell [23]. Changes in ZO-1 distribution and disruption of tight junction proteins, have been reported in hypoxia [24] and in oxygen glucose deprivation [20], resulting in increased EC permeability [25]. At the molecular level, ZO-1 changes can enhance permeability via tyrosine phosphorylation [6].

In malaria, ZO-1 reportedly plays an important role in the blood-brain barrier in cerebral malaria [26]. Destruction of EC-junction proteins--ZO-1, occludin and vinculin--in cerebral malaria has been reported [27]. An *in vitro* study of EC-PRBC co-culture showed decreased expression of tight junction RNAs from cerebral malaria patients. The study concluded that PRBCs can alter tight junction protein expression in ECs at the site of sequestration, and can influence disease severity [28]. The present study demonstrated that protein tight junction (ZO-1) was weakly detected in glomeruli and tubules. An additional study, using anti-ZO-1 to localize tight junction-associated protein, confirmed ZO-1 alteration and damage of the EC tight junction in ECs co-cultured with PRBCs. In the glomeruli, ZO-1 is localized in the capillary EC junction and between the foot processes. The structural alteration of the cell junction can increase cell permeability, leading to fluid and protein leakage. These changes cause further damage when coupled with hypovolaemia in malarial AKI. Further study of other junction and associated membrane proteins will help resolve the subcellular structural alteration responsible for permeability changes in severe malaria.

## Conclusions

Malarial AKI involves glomerular cell proliferation, despite reduced glomerular area. This study of tight junction-associated protein revealed a damaged tight junction structure (ZO-1) in severe *P. falciparum* malaria, associated with AKI. The ZO-1 damage in malarial AKI could contribute to the collapse of junction structures, thereby reducing glomerular area, which further increases cellular permeability. The finding suggests that AKI could contribute to ZO-1 damage and provide an insight into the pathogenesis of AKI in *P. falciparum* malaria infection.

## Competing interests

The authors declare that they have no competing interests.

## Authors’ contributions

BW focused on histopathology work and cell culture experiments, analysed the data, and drafted the manuscript. CP and UC performed the immunohistochemical work and helped analyse the data. PV formulated the research idea, designed the experiments and revised the final manuscript. All authors have approved the final version of this manuscript.
